# Human Papillomavirus Vaccination Status and Parental Endorsement Intentions among Undergraduate Student Nurses

**DOI:** 10.3390/ijerph18063232

**Published:** 2021-03-20

**Authors:** Ashley Hollins, Diane Wardell, Maria E. Fernandez, Christine Markham, Vincent Guilamo-Ramos, Diane Santa Maria

**Affiliations:** 1 Cizik School of Nursing, University of Texas Health Science Center at Houston (UTHealth), Houston, TX 77030, USA; Diane.wardell@uth.tmc.edu (D.W.); diane.m.santamaria@uth.tmc.edu (D.S.M.); 2 School of Public Health, The University of Texas Health Science Center at Houston (UTHealth), Houston, TX 77030, USA; Maria.E.Fernandez@uth.tmc.edu (M.E.F.); christine.markham@uth.tmc.edu (C.M.); 3 Center for Latino Adolescent and Family Health, New York University, New York, NY 10003, USA; Vincent.ramos@nyu.edu

**Keywords:** HPV vaccines, human papillomavirus vaccines, student nurses, papillomavirus vaccines, human papillomavirus viruses

## Abstract

We identified factors associated with student nurses’ Human Papillomavirus Vaccine (HPV) vaccination status and their intention to counsel parents on HPV vaccination. Undergraduate student nurses (*N* = 153) from a large university in the south participated. Descriptive statistics, chi-squared tests, and independent t-tests (*p* ≤ 0.05) were used to characterize the students’ vaccination status. Logistic regression was used to identify factors associated with vaccination status. HPV vaccination rates were low. Students who were older and married or living with a partner were less likely to have completed the HPV vaccine series. The most commonly cited reason for non-initiation and non-completion was the lack of provider endorsement. Vaccination status did not differ significantly according to race/ethnicity, religion, skills, or intention to counsel parents. While intentions to counsel parents on HPV vaccination are high among student nurses, interventions to improve vaccination rates among student nurses are needed.

## 1. Introduction

Human papillomavirus (HPV) is the most prevalent sexually transmitted infection (STI) in the United States [1]. Before vaccine introduction, an estimated 14 million new HPV infections occurred yearly, with nearly half occurring in persons aged 15–24 [1]. Persistent infection with high-risk HPV types may result in cervical, oropharyngeal, vulvar, vaginal, penile and anal cancer as well as genital warts and recurrent respiratory papillomatosis [2]. HPV-associated cancers have drastically increased, with 45,000 cases annually in the U.S. [3]. 

Sexually active adolescents and young adults have the highest prevalence of HPV infection, with 50–80% of cases occurring within 2–3 years of sexual debut [1]. It is estimated that approximately 3200 new cases of HPV-associated cancers are diagnosed in Texas each year [4]. The HPV-associated cancer incidence rate is lower in Texas (11.7 cases per 1000) when compared with the U.S. (12.3 cases per 100,000) [4]. However, HPV-associated cervical cancer incidence rate is significantly higher in Texas than in the U.S. [4]. In 2019, 48.4% of Texas adolescents aged 13 to 17 years old were up to date on the HPV vaccine, compared to the 54.2% national average [5].

The Advisory Committee on Immunization Practices (ACIP) recommends the HPV vaccine to 11- to 12-year-old adolescents, with catch-up vaccination for those aged 13–26 years [1]. The U.S. Food and Drug Administration (FDA) approved the use of the vaccine for women and men aged 27 through 45 years. [6]. Subsequently, the ACIP recommended shared clinical decision making regarding HPV vaccination for high-risk adults 27–45 years old who are not adequately vaccinated [1]. The HPV vaccine remains a cost-effective yet underutilized prevention strategy [7].

Although HPV affects all individuals, college-aged persons face substantially greater risks of HPV-related diseases than older adults; yet, many remain under-vaccinated [1]. In 2016, coverage with at least one dose of the HPV vaccine was only 48.5% among women and 13.5% among men aged 19–26 [8]. Studies indicate that vaccine uptake among college students is strongly associated with being younger [9], non-Hispanic [10], unmarried [11], and not practicing organized religion [10]. Studies also indicate that a provider’s recommendation [12], supportive HPV vaccine beliefs of peers and significant others [9], and low vaccination costs [13] are key determinants to vaccine uptake among college students. 

College-aged youth are a priority group for HPV catch-up vaccinations [14]. Existing research among 18- to 26-year olds has focused on investigating the determinants of vaccine uptake including HPV vaccine knowledge, attitudes and beliefs, and provider recommendation [15]. Since the HPV vaccination recommendations were established, few studies have examined the factors associated with HPV vaccination status among college students, and no research to date on HPV vaccine uptake has focused specifically on student nurses despite their future role in vaccination endorsement and delivery. 

Student nurses, as future professionals in the largest segment of the health care system, are uniquely positioned to provide vaccine education, address parental concerns, and offer strong vaccine endorsements. Research has shown that nurse-led vaccine promotion improves uptake [16,17]. Studies suggest that nurses with positive attitudes toward vaccination and who express a willingness to be vaccinated are more likely to recommend HPV vaccinations [18,19]. Thus, identifying the factors associated with HPV vaccine uptake may help address student nurses’ personal HPV vaccine uptake and completion and their intention to provide a strong HPV vaccine endorsement.

### Study Aims

The primary aim of this study was to identify factors associated with student nurses’ HPV vaccination status, their preferred counseling strategies, perceptions of parental vaccination hesitancy, and to assess whether their skills and intentions to counsel parents differed by their vaccination status. We assessed student nurses’ HPV vaccination status in a diverse sample of student nurses recruited from a large urban nursing school in the southwest. 

## 2. Materials and Methods

### 2.1. Design

The data in this study were derived from the baseline survey of a large randomized controlled intervention trial. Undergraduate student nurses were recruited between 2015 and 2018. 

### 2.2. Data Collection

Data collection began after approval from the university’s Institutional Review Board. Survey items were drawn from measures used in previous published studies of HPV vaccination uptake [20,21], skills satisfaction [22], and HPV-endorsement outcome expectations [7]. 

### 2.3. Measures 

Demographics pertaining to age, race/ethnicity, marital status, and religion were collected. The students’ vaccination status, including vaccination non-initiation, initiation, and completion, were assessed. Non-initiation was defined as not receiving any HPV dose. Initiation was defined as receiving at least one dose of HPV vaccine. Completion was defined as having completed all three doses of the HPV vaccination series (the recommended dosing at the time of this study). 

Factors associated with vaccination status were assessed using questions adapted from Kessel et al. [20]. Participants were asked about their current level of confidence in providing counseling and education to parents and youth using 14-items developed by Guilamo-Ramos et al. (α = 0.65) [22]. Participants were also asked to rate their level of satisfaction as both parent-communication counselors and adolescent health educators using a 5-point Likert scale. 

The HPV-endorsement outcome expectation scale was used to assess students’ beliefs about HPV vaccine endorsement; using an eight-item scale developed by McRee, Gilkey, and Dempsey [7]. Students also reported their beliefs about HPV vaccine endorsement. 

Intention to counsel parents on HPV vaccination was assessed via two items developed by investigators. Perceived parental barriers to HPV vaccine uptake and preferred counseling strategies were assessed using items from the Minnesota Health Care Provider Survey [7]. 

### 2.4. Analytic Strategies

Data were analyzed using IBM Statistical Package for Social Sciences (SPSS, version 25, IBM, Armonk, NY, USA). Descriptive statistics were used to analyze demographic distribution and to stratify the participants by vaccination status. *p* values of less than 0.05 were considered statistically significant for all analyses. Bivariate frequencies and chi-squared tests were then calculated to compare HPV vaccination status by demographic variables. Statistically significant variables were included in the final multivariable analyses of HPV vaccination initiation and completion. Bivariate associations of age and marital status with HPV vaccination completion were examined using logistic regression and were expressed as unadjusted odds ratios (OR). For the logistic regression analyses, HPV vaccination completion was dichotomized. The relations between age, marital status, and HPV vaccination completion were then examined using a multivariable logistic regression model. 

Univariate descriptive statistics were computed to describe the distribution of each outcome variable. Next, independent-samples *t*-tests were used to determine the mean differences between non-initiators and initiators and between initiators and completers. The rest is on your table. Finally, we examined differences in intentions to counsel parents on HPV vaccination status between non-initiators and initiators and between initiators and completers. The Mann–Whitney U test was used due to a non-normal distribution of the data.

## 3. Results

### 3.1. Sample Characteristics

The mean age of participants (*N* = 153) was 24.5 years (SD = 5.95), with 73% (*n* = 112) aged between 18 and 26 years. The majority of participants were non-Hispanic (*n* = 111, 73%) and self-identified their race as either Caucasian (*n* = 97, 63.4%), Asian (*n* = 23, 15%), African American (*n* = 15, 9.8%) or Other (*n* = 18, 11.8%). Nearly three-fourths of the participants (*n* = 107, 70%) were single, with 30% (*n* = 46) stating that they were married or living with a partner. The vast majority of the participants identified as religious (*n* = 133, 87%); 73% (*n* = 111) self-identified as Christian (Table 1). 

### 3.2. HPV Vaccination Status 

Forty-two percent (*n* = 64) of the students reported that they had not initiated HPV vaccination (Table 1). Of students who had initiated the vaccine, 42% (*n* = 65) reported that they had received all three doses of the HPV vaccine. Caucasian (*n* = 56, 62.9%) and Hispanic (*n* = 29, 32.5%) participants were more likely to initiate the vaccine than Asian (*n* = 13, 14.6%) or African American (*n* = 10, 11.2%) students. Caucasian and Hispanic students had higher rates of HPV vaccination completion (*n* = 40, 61.5% and *n* = 19, 29%, respectively) than Asian (*n* = 9, 13.8%) and African American (*n* = 9, 13.8%) students.

Chi-squared tests indicated a significant relationship between HPV vaccination completion and marital status (X^2^ {3, *n* = 153} = 20.11, *p* < 0.000) among those who had received at least one dose of HPV vaccine (initiators). Specifically, married students were less likely to have received the HPV vaccine than were single students. In addition, the relationship between vaccination completion and age was significant (*t* {151} = 4.36, *p* = 0.00) among completers and non-completing initiators; older students were less likely to have completed the HPV vaccine series than younger students. The percentage of students who were non-completing initiators and completers did not differ by race (X^2^ {4, *N* = 147} = 3.55, *p* > 0.47), ethnicity (X^2^ {1, *N* = 153} = 0.18, *p* > 0.67), or religion (X^2^ {8, *N* = 152} = 7.83, *p* > 0.45) (data not shown). Therefore, only age and marital status were included in the logistic regression. 

To further explore vaccination completion, a binary logistic regression model was conducted among non-completing initiators and completers. In the regression model, marital status as well as age were significantly associated with vaccination completion. Students who identified as single (OR: 2.65; 95% CI: 1.09–6.42, *p =* 0.031) were almost three times more likely to have completed HPV vaccination than married students. Age (OR: 0.86; CI: 0.78–0.95; *p =* 0.003) was significantly associated with a decreased likelihood of vaccination completion; an increase of one year in age was associated with a 14% reduction in the odds of vaccination completion. The Hosmer–Lemeshow fit statistics indicated an acceptable model fit (χ^2^ = 8.50, *p =* 0.39) (Table 2).

Students who had received at least one HPV vaccine dose (*n* = 89) reported that the most important factors influencing vaccination initiation were a provider’s recommendation (*n* = 25, 39%), confidence in the HPV vaccine (*n* = 24, 27%), having a parent who recommended the vaccine (*n* = 9, 14%), and having adequate knowledge of HPV disease and the vaccine (*n* = 9, 14%). The most commonly cited factors that influenced vaccination completion were age (<26) (*n* = 21, 24%), a provider’s recommendation (*n* = 17, 19%), and the convenience of receiving the vaccine (*n* = 15, 17%). The most common reasons for not completing the HPV series among non-initiators and non-completing initiators were age eligibility (>26 years) (*n* = 17, 26%), lack of provider recommendation (*n* = 14, 16%), having an intention to complete the HPV series but not having done it yet (*n* = 12, 14%), and not being sexually active (*n* = 7, 8%). 

Overall baseline scores for HPV-endorsement outcome expectations, skills satisfaction, and counseling intentions were high among the total sample. Independent-samples *t*-tests found no statistically significant differences in the scores for mean HPV-endorsement outcome expectations, skills satisfaction as a parent sexual-health communication counselor, or skills satisfaction as an adolescent sexual-health educator between non-initiators and initiators or between initiators and completers (Table 3). No significant difference in the intention to counsel parents on the HPV vaccination was found between non-initiators and initiators (*U* = 2661, *Z* = −0.952, *p =* 0.341) or between vaccination initiators and completers (*U* = 2777, *Z* = −0.419, *p = 0.675*) (Table 4). 

Most students perceived that the factors contributing to parental decisions included parents’ beliefs that their children are not sexually active (*n* = 73, 57%) and their discomfort in talking with their children about sex (*n* = 64, 53.1%). Perceived factors that contributed “a little” or “not at all” to parental decisions to delay or refuse HPV vaccination for their child included the parents’ belief that the HPV vaccine is not very effective (*n* = 99, 77.3%), that parents let their children decide and the children refuse the vaccine (*n* = 99, 77.3%), and that the child was sick at the time of the visit (*n* = 101, 78.9%). In addition, student’s perception that parental concern that their children would suffer immediate short-term effects (*n* = 94, 73.4%) and/or long-term complications from the HPV vaccine (*n* = 94, 73.4) contributed “a little” or “not at all” to parental delay or refusal of the HPV vaccine. 

### 3.3. Tools and Strategies for Counseling HPV Vaccine-Hesitant Parents

Students perceived that the following tools and strategies would be most helpful in counseling parents who hesitate to vaccinate their children for HPV: information tailored to the patient’s cultural background (*n* = 122, 87%) and specific parental concerns (*n* = 125, 89%) and providing information separately to parents and adolescents (*n* = 106, 76%). Participants perceived that providing information to parents about the HPV vaccine prior to the clinical visit (*n* = 102, 73%) and giving interactive decision aids to prioritize their health values (*n* = 113, 81%) would also be beneficial when counseling parents. 

## 4. Discussion

This study is among the first to examine the prevalence of HPV vaccination initiation and completion among a diverse sample of student nurses. Student nurses represent the next generation of the largest segment of health care providers and are in a position to influence vaccination uptake and completion with strong and effective vaccination endorsement. As HPV-associated cancer rates steadily rise and vaccine uptake remains low, it is crucial to identify factors that could further our efforts to prevent HPV-related sequela. 

This study’s findings indicate that HPV vaccination initiation remains low despite national initiatives to improve HPV vaccination rates. Additionally, vaccine completion rates were far below the Healthy People 2030 goal of 80% coverage. These rates mirror rates in previous studies of college students which range from 25 to 63% [23,24,25,26] and completion (27–60.7%) [27]. These findings also align with recent national estimates of initiated HPV vaccination [8]. Continued efforts are urgently needed to improve catch-up vaccination rates and eliminate missed vaccination opportunities among college students, including student nurses, particularly in light of new ACIP recommendations for regarding HPV vaccination for those aged 27–45 [1]. One way to do this is to actively promote HPV vaccination on college campuses through student health programs [12]. Additionally, colleges and universities should recommend the HPV vaccination to all student nurses [28].

The majority of the participants in this study were young women. At the time of this study, the HPV vaccine was recommended for women through age 26, men through age 21, and through age 26 for men who have sex with men, identify as gay or bisexual, or intend to have sex with men, and young adults who identify as transgender or who are immunocompromised (including HIV) [29]. Of those over the age-eligibility recommendations at the time of this study, many expressed interests in receiving the vaccine. For those adults, it is not too late to get vaccinated as the FDA recently expanded the use of Gardasil 9 HPV vaccine to all women and men through age 45 [6]. Findings from this study coupled with expanded recommendation age suggest a need for enhanced outreach efforts to educate student nurses, adults under age 45, and providers about the expanded HPV vaccination recommendation.

While the number of young adults receiving the HPV vaccine has increased over the last several years, there are still disparities by race/ethnicity [30]. In this study, we found that the proportion of students who had initiated vaccination in the current sample differed by race/ethnicity, with Caucasians being more likely to initiate and complete the HPV series than other racial/ethnic groups. These findings are similar to national estimates, which recently showed that Caucasians (adults aged > 19) (48.5%) are more likely to initiate the HPV vaccination than African Americans (37.7%) and young Hispanic adults (33%) [31]. However, one study found that counties with large African American populations had higher levels of HPV vaccination uptake than those that were predominately Caucasian [32]. Studies report that ethnic minority populations accept vaccination, but cultural differences may create barriers to vaccine uptake [33] as well as lack of access and availability of health care services [34] and more specifically, HPV vaccination [9]. Therefore, it is important to develop culturally relevant education and service delivery models that may increase HPV vaccine uptake and completion among minority groups.

In our study, we found that higher vaccination completion was associated with being unmarried. This may be related to a greater perceived risk of HPV infection among unmarried student nurses. Those reported being married or living with a partner were less likely to have completed vaccination. This may be due to a belief that they are not at risk of contracting STIs. The literature suggests that single women were significantly more likely than married women to be interested in HPV vaccination [11]. Therefore, relationship status may influence HPV vaccination by impacting risk perceptions and perceived need. Thus, efforts to improve HPV vaccination rates should address the lifetime risk of acquiring HPV among young adults in relationships.

Additionally, provider’s recommendation was the most influential factor associated with vaccine initiation and completion among student nurses. One study found that young adults who received a provider’s recommendation were over 35 times more likely to receive at least one dose of HPV vaccine than those who received no recommendation [35]. Our study highlights that health care providers play an important role in health promotion and disease prevention. The lack of provider recommendation was cited as a reason for non-initiation and non-completion of the HPV vaccine series among under-vaccinated students in this study. Failure to discuss vaccination may result in missed opportunities during routine physicals may ultimately lead to the development of more HPV-related sequela. Therefore, provider education on the expansion of the recommended age for HPV vaccination is critical to assuring student nurses, and all adults up to age 45, are being offered the HPV vaccine [1]. 

Differences in vaccination status associated with the students’ intentions to counsel, their outcome expectations, and their skills satisfaction as both a sexual-health parent-communication counselor and as an adolescent sexual-health educator were also assessed. Surprisingly, there was no significant difference between the two groups (non-initiators vs. initiators and initiators vs. completers). These findings suggest that vaccination status did not have a negative or positive impact the students’ beliefs about counseling, skills satisfaction, outcome expectations, or intentions to counsel parents about the HPV vaccination. This may be due the low variability in the overall sample scores on skills satisfaction, outcome expectations, and intentions to counsel parents about the HPV vaccination which were high among the total student nurses [36,37].

While there is some evidence that a nurse’s personal beliefs and self-confidence in addressing vaccine concerns drive their vaccine-endorsement practices [38,39], we did not find such a connection among student nurses. This is important since studies continue to find a lag in provider endorsement for Hispanic boys [40] and that highly educated, affluent, and Caucasian parents may be less likely to opt into HPV vaccination for their children [41]. Intentions to endorse the HPV vaccination with parents was high. However, vaccination rates among student nurses were low. Further research is needed to determine if improving vaccination rates among nurses will improve HPV vaccination endorsement and to address the low vaccination rates of future providers. Studies suggest that nurses who are willing to be vaccinated and have a positive attitude toward vaccination and are more likely to recommend HPV vaccination to their patients [18,19].

Our findings indicate that the student nurses perceived parental barriers—such as the belief that their child is not sexually active, discomfort in talking with their child about sex, and the belief that their child was unlikely to get an HPV-related disease—as factors that impact HPV vaccination rates. This is consistent with the previous literature, which suggests that factors such as reluctance to discuss sexual health, a child’s age, and a perception of low risk are common barriers to HPV vaccine uptake and completion among patients [7,42]. 

## 5. Limitations and Strengths

The limitations of this study include the use of previously collected data and self-reported measures without confirmatory HPV titers or confirmation of vaccination records. Self-reported measures (i.e., intention to counsel parents) may reflect what student nurses felt to be the most desirable answer and may not indicate their actual future practices. Additionally, participants were recruited using a purposive sample of student nurses from one Bachelor of Science nursing school in an urban area, which may limit the generalizability of the findings. While data on gender were not collected, it is important to note that most student nurses were female. Gender differences in vaccination rates are well documented, with females reporting higher HPV vaccination rates [43]. 

Despite these limitations, the focus on student nurses is an innovative approach to deepening our understanding of the HPV vaccination status of student nurses and of the barriers to and facilitators of vaccine endorsement. This research provides insight on student nurses, who are transitioning to a nursing role in which they will have the opportunity to promote HPV vaccination and to reduce missed opportunities for HPV prevention. This study’s findings provide direction for future programs and interventions aimed at young adults and emerging health care providers that address vaccination concerns and may increase awareness, HPV vaccine uptake, and vaccination completion. 

## 6. Conclusions

Given the growing epidemic of HPV infection, more research is needed, particularly among high-risk populations and future health care providers that may increase vaccine-promoting behaviors and impact clinical practice. Although the current acceptance of HPV vaccination is promising, uptake and completion rates remain low among college students, specifically student nurses. Therefore, it is important to develop targeted interventions to increase vaccination rates among student nurses to protect them from HPV-related diseases. 

Student nurses are uniquely positioned to promote HPV vaccination acceptance and completion despite their own personal vaccine status. 

## Figures and Tables

**Table 1:** Sample characteristics by HPV vaccination status among student nurses (*n* = 153).

Variables	Non-Initiators (*n* = 64)	Initiators (*n* = 89)		Total (*n* = 153)
	Non-Initiators (*n* = 64); *n* (%)	Non-Completing Initiators (*n* = 24); *n* (%)	Completers (*n* = 65); *n* (%)	*n* (%)
Demographics				
Age in years, M (SD) †				24.5 (5.95)
≤26, *n* (%)	36 (56)	20 (83)	56 (86)	112 (73.2)
>26, *n* (%)	28 (44)	4 (17)	9 (14)	41 (26.8)
Ethnicity, *n* (%)				
Hispanic	13 (20)	10 (42)	19 (29)	42 (27.4)
Non-Hispanic	51 (80)	14 (58)	46 (71)	111 (72.6)
Race, *n* (%)				
Caucasian	41 (64)	16 (66.6)	40 (61.5)	97 (63.4)
Asian	10 (15.6)	4 (16.6)	9 (13.8)	23 (15)
African American	5 (7.8)	1 (4.1)	9 (13.8)	15 (9.8)
Other ‡	8 (12.5)	3 (12.5)	7 (10.8)	18 (11.8)
Marital status, *n* (%)				
Single/Not in a relationship	33 (52)	18 (75)	56 (86)	107 (70)
Married/Living with a partner	31 (48)	6 (25)	9 (14)	46 (30)
Religion, *n* (%)				
Christian	45 (70)	17 (70.8)	49 (75.3)	111 (72.6)
Other §	19 (30)	7 (29)	16 (25)	42 (27.4)

Note: † Mean (standard deviation); ‡ American Indian or Native American; multiracial; something else; § Atheist, Buddhist, Hindu, Jewish, Muslim, none of the above, refuse to answer.

**Table 2:** Factors Associated with Vaccination Completion (Completers vs. All Other Groups) among Student Nurses.

	Coefficient B	SE †	OR ‡	*p* Value	95% CI §
Age	−0.149	0.050	0.86	0.003	0.78–0.95
Marital Status	0.975	0.452	2.65	0.031	1.09–6.42

Note: † SE: standard error; ‡ OR: odds ratio; § CI: confidence interval of the odds ratio.

**Table 3:** Independent-samples *t*-test comparing non-initiators and initiators.

Variable	Group	*N*	Mean	Std. Deviation	Std. Error Mean	*t*	Sig. (2-Tailed)
Skills Satisfaction as a Sexual-Health Parent-Communication Counselor	Non-Initiators	64	8.94	3.984	0.498	1.047	0.297
	Initiators	89	9.62	3.956	0.419		
Skills Satisfaction as an Adolescent Sexual-Health Educator	Non-Initiators	64	25.70	8.295	1.037	1.140	0.256
	Initiators	89	27.30	8.748	0.927		
HPV-Endorsement Outcome Expectations	Non-Initiators	56	17.23	4.138	0.553	0.288	0.774
	Initiators	78	17.44	3.966	0.449		
Independent-Samples *t*-Test Comparing Initiators and Completers							
Skills Satisfaction as a Sexual-Health Parent-Communication Counselor	Initiators	88	9.50	4.283	0.457	0.603	0.547
	Completers	65	9.11	3.518	0.436		
Skills Satisfaction as an Adolescent Sexual-Health Educator	Initiators	88	26.99	8.880	0.947	0.594	0.553
	Completers	65	26.15	8.176	1.014		
HPV-Endorsement Outcome Expectations	Initiators	77	17.32	3.995	0.455	−0.87	0.931
	Completers	57	17.39	4.100	0.543		

**Table 4:** Mann–whitney *U* test comparing non-initiators and initiators.

Variable	Group	*N*	Mean Rank	Sum of Ranks	*U* †	*Z* ‡	*p* Value
Intentions to Counsel Parents on HPV Vaccination	Non-Initiator	64	74.08	4741	2661	−0.952	0.341
	Initiator	89	79.10	7040			
	Total	153					
Intentions to Counsel Parents on HPV Vaccination	Initiator	88	76.06	6693.50	2777.500	−0.419	0.675
	Completer	65	78.27	5087.50			
	Total	153					

Note: † *U*: Mann–Whitney; ‡ *Z*: *Z* value.

## Data Availability

Please contact the corresponding author to request the data supporting reported results of this study.
